# Ultrasound Diagnosis of a Cervical Rib with Pseudarthrosis

**DOI:** 10.5334/jbsr.2661

**Published:** 2021-12-29

**Authors:** Douglas Lacomblez, Dana Dumitriu

**Affiliations:** 1 Cliniques Universitaires Saint-Luc, BE

**Keywords:** cervical rib, ultrasound, neck, lump, pseudarthrosis

## Abstract

**Teaching Point:** Ultrasound is the first-line examination for neck mass and may offer a reliable method to identify cervical ribs.

## Case

A 13-year-old girl was referred for a painless rigid mass in the right supraclavicular fossa, present for several weeks. There were no other systemic complaints, nor vascular or neurological symptoms in the right upper limb. There was no history of trauma or other significant medical history.

**Figure 1:**
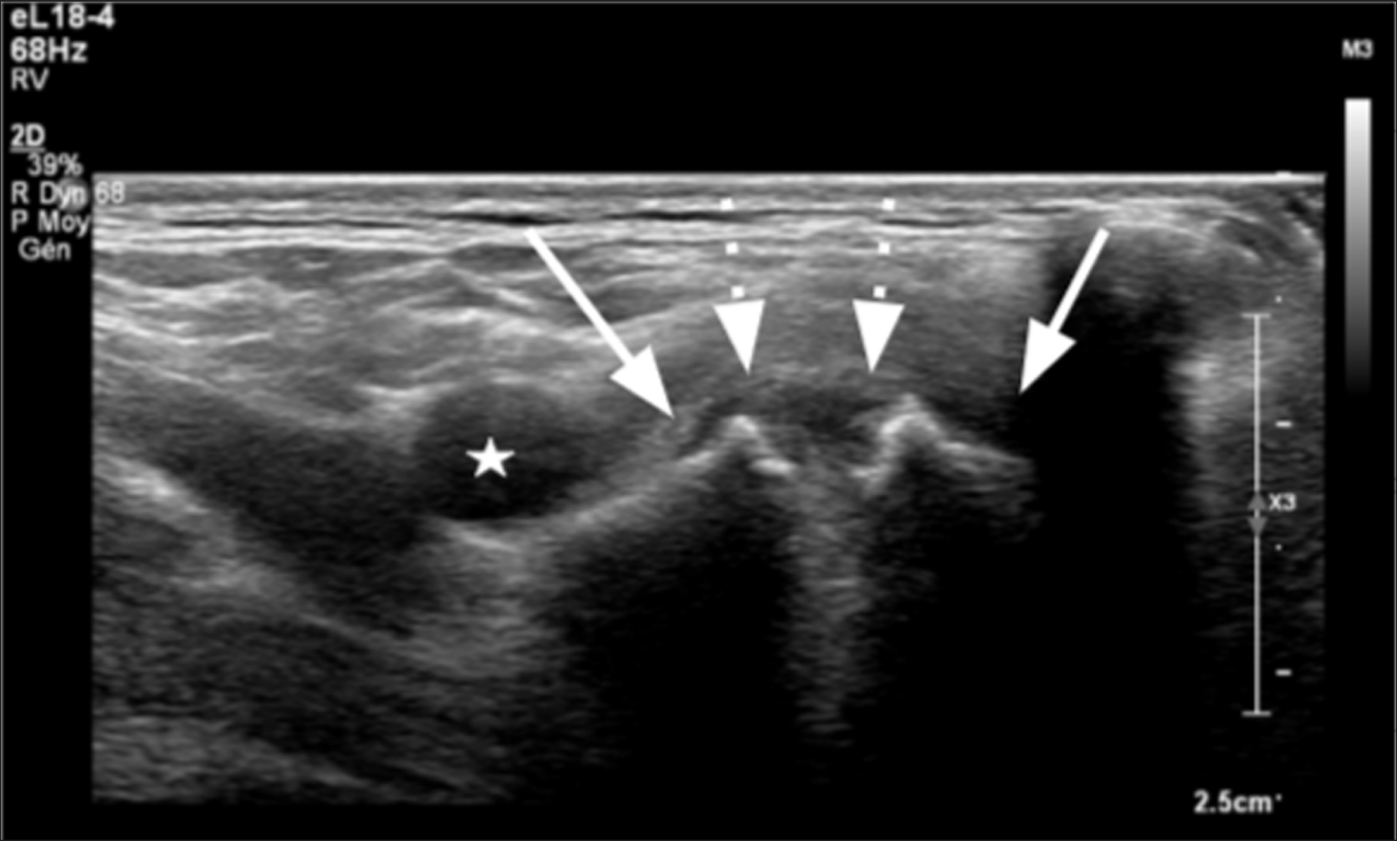


Ultrasound (US) of the right supraclavicular region was performed with high frequency L18-4 MHz linear probe (Philips Healthcare, Eindhoven, Netherlands). It showed two linear echoic bone structures (***Figure 1***, plain arrow) with a roundish hypo-echoic area that resembled cartilage in between (***Figure 1***, dotted arrow), connecting to form a joint-like structure, corresponding to the lump. The right subclavian artery was identified, coursing upward and laterally from this structure (***Figure 1***, white star).

**Figure 2:**
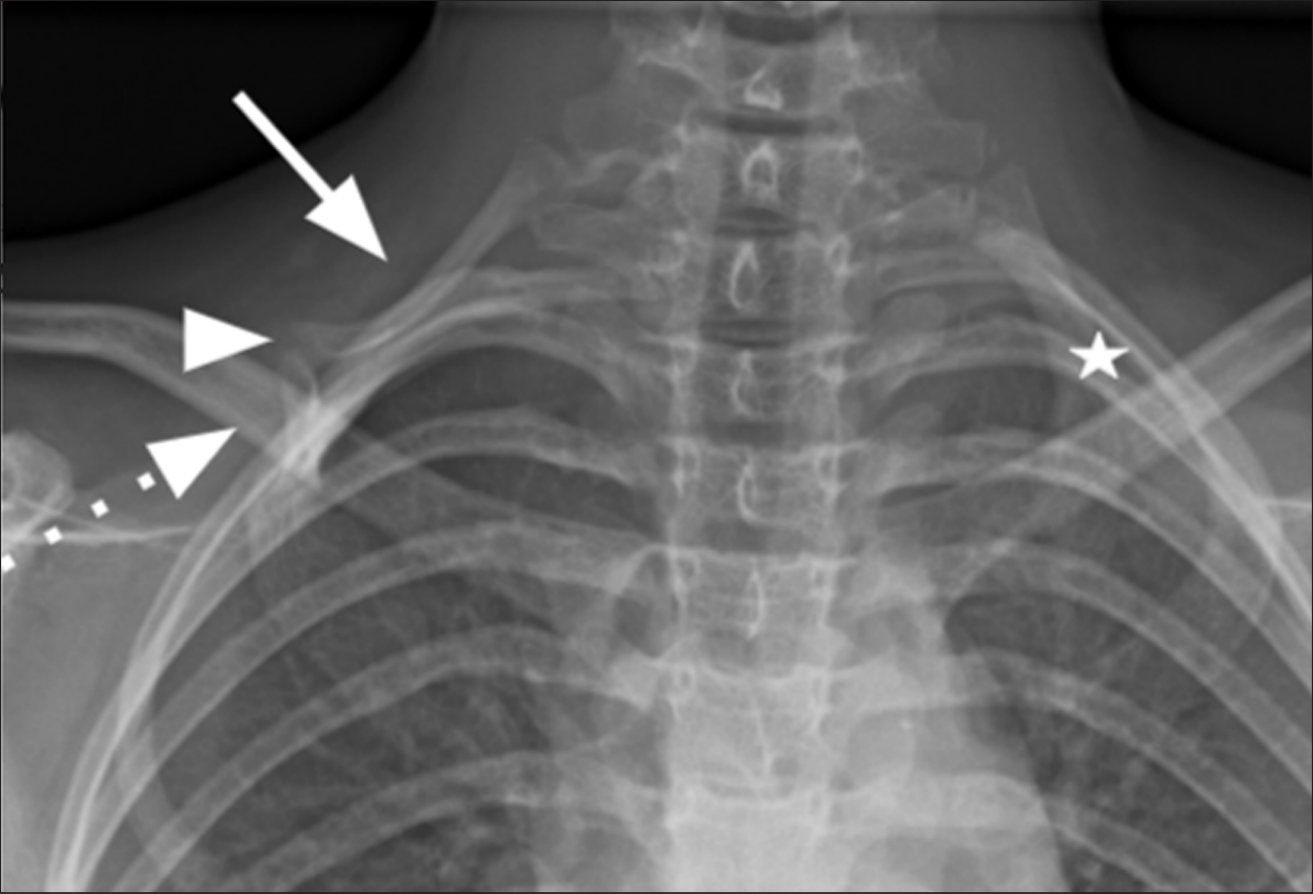


Cervical and comparative X-rays of the clavicular region were performed, showing a right complete cervical rib (***Figure 2***, plain arrow), forming a pseudarthrosis (***Figure 2***, arrowhead) with a bony protrusion from the lateral arch of the first right rib (***Figure 2***, dotted arrow) A shorter left cervical rib was also present (***Figure 2***, white star).

## Comment

Prevalence of cervical ribs in healthy individuals varies from 0.58% to 6.2%, with a slight female predominance and 75.8% bilateral occurrence in the pediatric population [1].

Cervical ribs may cause symptoms related to vascular compression, in particular when the rib extends beyond the transverse process of the vertebra, articulating with the first rib. Although this was the case in our patient, she had no vascular complaints. Conversely, shorter cervical ribs are prone to neurological symptoms due to compression of the inferior trunk of the brachial plexus [1].

Ultrasound is the first-line diagnostic tool to evaluate neck masses in children. A palpable neck mass in a child is a common indication for imaging, and more ominous diagnoses, such as sarcoma or lymphoma, need to be excluded. Knowledge of anomalies associated with cervical ribs and their presentation on US may avoid the use of more complex or more costly imaging techniques, such as MRI or CT.

In this case, a soft-tissue mass, lymph node, or vascular anomaly was immediately excluded with US and cervical rib suspected. The detailed description of the bone malformation was further refined with X-ray.
